# Cerebrospinal fluid biomarker supported diagnosis of Creutzfeldt–Jakob disease and rapid dementias: a longitudinal multicentre study over 10 years

**DOI:** 10.1093/brain/aws238

**Published:** 2012-09-25

**Authors:** Katharina Stoeck, Pascual Sanchez-Juan, Joanna Gawinecka, Alison Green, Anna Ladogana, Maurizio Pocchiari, Raquel Sanchez-Valle, Eva Mitrova, Theodor Sklaviadis, Jerzy Kulczycki, Dana Slivarichova, Albert Saiz, Miguel Calero, Richard Knight, Adriano Aguzzi, Jean-Louis Laplanche, Katell Peoc’h, Gabi Schelzke, Andre Karch, Cornelia M. van Duijn, Inga Zerr

**Affiliations:** 1 Department of Neurology, National Reference Centre for Transmissible Spongiform Encephalopathies, Georg-August-University, 37075 Göttingen, Germany; 2 Department of Neurology, University Hospital Marqués de Valdecilla, Fundación Marqués de Valdecilla IFIMAV and Centro de Investigación Biomédica en Red sobre Enfermedades Neurodegenerativas (CIBERNED), 39008 Santander, Spain; 3 National CJD Surveillance Unit, University of Edinburgh, EH4 2XU, UK; 4 Department of Cell Biology and Neurosciences, Istituto Superiore di Sanità, 00161 Rome, Italy; 5 Department of Neurology, Creutzfeldt–Jakob Disease Unit, Hospital Clínic, 08036 Barcelona, Spain; 6 Institute of Preventive and Clinical Medicine, Slovak Medical University, 83301 Bratislava, Slovakia; 7 Department of Pharmaceutical Sciences, Laboratory of Pharmacology, School of Health Sciences, Aristotle University of Thessaloniki, EL 54124, Greece; 8 I-st Neurological Department, Institute of Psychiatry and Neurology, 02-957 Warsaw, Poland; 9 National Centre of Microbiology, Carlos III Health Institute, 28220 Madrid, Spain; 10 Institute of Neuropathology, University Hospital, 8091 Zurich, Switzerland; 11 UF de Génétique Moléculaire, Laboratoire associé au CNR ‘ATNC’, Hôpital Lariboisiere, 75010 Paris, France; 12 Departments of Epidemiology and Biostatistics, Erasmus University Medical Center, 3000 DR Rotterdam, The Netherlands

**Keywords:** rapid dementia, Creutzfeldt–Jakob disease, cerebrospinal fluid, 14-3-3, specificity, neurodegeneration, differential diagnosis in dementia

## Abstract

To date, cerebrospinal fluid analysis, particularly protein 14-3-3 testing, presents an important approach in the identification of Creutzfeldt–Jakob disease cases. However, one special point of criticism of 14-3-3 testing is the specificity in the differential diagnosis of rapid dementia. The constant observation of increased cerebrospinal fluid referrals in the national surveillance centres over the last years raises the concern of declining specificity due to higher number of cerebrospinal fluid tests performed in various neurological conditions. Within the framework of a European Community supported longitudinal multicentre study (‘cerebrospinal fluid markers’) we analysed the spectrum of rapid progressive dementia diagnoses, their potential influence on 14-3-3 specificity as well as results of other dementia markers (tau, phosphorylated tau and amyloid-β_1–42_) and evaluated the specificity of 14-3-3 in Creutzfeldt–Jakob disease diagnosis for the years 1998–2008. A total of 29 022 cerebrospinal fluid samples were analysed for 14-3-3 protein and other cerebrospinal fluid dementia markers in patients with rapid dementia and suspected Creutzfeldt–Jakob disease in the participating centres. In 10 731 patients a definite diagnosis could be obtained. Protein 14-3-3 specificity was analysed for Creutzfeldt–Jakob disease with respect to increasing cerebrospinal fluid tests per year and spectrum of differential diagnosis. Ring trials were performed to ensure the comparability between centres during the reported time period. Protein 14-3-3 test specificity remained high and stable in the diagnosis of Creutzfeldt–Jakob disease during the observed time period across centres (total specificity 92%; when compared with patients with definite diagnoses only: specificity 90%). However, test specificity varied with respect to differential diagnosis. A high 14-3-3 specificity was obtained in differentiation to other neurodegenerative diseases (95–97%) and non-neurological conditions (91–97%). We observed lower specificity in the differential diagnoses of acute neurological diseases (82–87%). A marked and constant increase in cerebrospinal fluid test referrals per year in all centres did not influence 14-3-3 test specificity and no change in spectrum of differential diagnosis was observed. Cerebrospinal fluid protein 14-3-3 detection remains an important test in the diagnosis of Creutzfeldt–Jakob disease. Due to a loss in specificity in acute neurological events, the interpretation of positive 14-3-3 results needs to be performed in the clinical context. The spectrum of differential diagnosis of rapid progressive dementia varied from neurodegenerative dementias to dementia due to acute neurological conditions such as inflammatory diseases and non-neurological origin.

## Introduction

The clinical diagnosis of Creutzfeldt–Jakob disease is based on clinical syndrome and results of established paraclinical tests (EEG, CSF analysis and cranial MRI) (Zerr *et al.*, 2000*a*, 2009; Collins *et al.*, 2006). The predominant clinical symptoms are characterized by rapid progressive dementia followed by development of neurological signs, e.g. ataxia and myoclonus (Gambetti *et al.*, 2003). The disease is fatal and leads to death in a few months.

Detection of periodic sharp wave complexes on EEG substantiated the diagnosis of sporadic Creutzfeldt–Jakob disease for a long period of time (Masters *et al.*, 1979; Zerr *et al.*, 2000*a*, *b*). From 1995 onwards, detection of neuronal destruction markers in CSF have become more and more important. They have been established in diagnostic work-up, with protein 14-3-3 as the most promising surrogate marker finally included in the WHO criteria (Hsich *et al.*, 1996; Zerr *et al.*, 2000*a*). Because elevated levels of protein 14-3-3 are also reported in a range of non-prion-related diseases, mostly caused by an acute neurological event such as encephalitis, stroke, epileptic fit or tumour, positive results must be interpreted in the clinical context.

Another major step in the diagnosis of sporadic Creutzfeldt–Jakob disease was the introduction of MRI, where a specific pattern in patients with sporadic Creutzfeldt–Jakob disease, characterized by hyperintense signals in basal ganglia and cortical regions, was identified (Tschampa *et al.*, 2007; Meissner *et al.*, 2008). Especially sensitive MRI techniques such as FLAIR and diffusion-weighted MRI sequences allow a diagnosis of sporadic Creutzfeldt–Jakob disease with high sensitivity and specificity (Satoh *et al.*, 2007; Meissner *et al.*, 2009). As a result, a positive MRI scan was proposed to be included into the clinical criteria in 2009 (Zerr *et al.*, 2009).

To date, CSF analysis, in particular protein 14-3-3 testing, presents an important approach in the identification of Creutzfeldt–Jakob disease cases and a request for CSF testing to specialized laboratories is frequently a major way to obtain referrals to surveillance centres. However, one special point of criticism of 14-3-3 testing was a potential loss of specificity and several reports on that have been published (Chohan *et al.*, 2010; Coulthart *et al.*, 2011; Perry and Geschwind, 2011). Within the framework of a European Community (EC) supported longitudinal multicentre study (‘CJD markers’) we evaluated the specificity of 14-3-3 in Creutzfeldt–Jakob disease diagnosis from the years 1998–2008. In addition we analysed the spectrum of rapid progressive dementia diagnoses, their potential influence on 14-3-3 specificity as well as results of related dementia markers (tau, phosphorylated tau, amyloid β_1–42_) in different forms of rapid dementias. We present results of regular ring trials conducted in participating surveillance laboratories to analyse inter-laboratory reliability (Supplementary material).

The term ‘rapid progressive dementia’ or shortened ‘rapid dementia’ we used in this study summarizes a condition of cognitive deterioration that can be attributed to either a neurological (most commonly neurodegenerative) or non-neurological disease (Geschwind *et al.*, 2008). Potentially reversible conditions (e.g. acute delirium, pseudo-dementia in depression, CNS inflammation) can appear frequently and need to be considered in the diagnostic process.

## Materials and methods

### Patients

The study was conducted within the framework of an EC-supported multinational study (‘CJD markers’) (Sanchez-Juan *et al.*, 2006).

Patients were referred to national surveillance units for detection of CSF 14-3-3 protein during the course of routine clinical diagnosis and surveillance. Samples were sent to the individual laboratories for the detection of CSF 14-3-3, further for CSF tau, phosphorylated tau and amyloid β_1–42_ in participating countries between 1998 and 2008. All countries collected clinical and neuropathological data from those patients with a clinical suspicion of Creutzfeldt–Jakob disease or related disorders. The corresponding diagnoses were obtained by follow-up.

### Patient’s data collection and analysis

Clinical data such as age at onset, gender, disease duration, time point of lumbar puncture, final clinical and neuropathological diagnoses were collected by each centre. A database was set up, which included detailed data on the CSF markers and patients’ characteristics for a total of 29 022 samples from patients with Creutzfeldt–Jakob disease and non-Creutzfeldt–Jakob disease (other rapid dementia) diagnoses. In 10 731 patients, a definite diagnosis could be obtained. From the remaining 18 291 samples, no further clinical data were available. In all participating centres, the diagnosis of Creutzfeldt–Jakob disease was made according to established, internationally agreed criteria (WHO, 1998; Zerr *et al.*, 2009).

Control patients were those who proved an alternative diagnosis or in whom Creutzfeldt–Jakob disease was definitely excluded on clinical or pathological grounds. Diagnoses in control patients were based on neuropathology (whenever possible) and clinical follow-up information.

For calculation of 14-3-3 specificity we used a two-step approach: (i) analysis was restricted to patients with a definite diagnosis (*n=* 10 731) and (ii) total specificity was calculated using all available samples. When a clinical diagnosis was available, patients were assigned to one of the following groups: neurodegenerative, inflammatory, CNS tumour/paraneoplastic, stroke, psychiatric, metabolic and ‘other’ (Table 1).

**Table 1:** Diagnostic spectrum of rapid dementia in CSF biomarkers from patients with definite clinical/neuropathological diagnosis

Diagnosis	*n* (%)
Sporadic Creutzfeldt–Jakob disease	3254 (30.3)
Genetic Creutzfeldt–Jakob disease	229 (2.1)
Iatrogenic Creutzfeldt–Jakob disease	46 (0.4)
Variant Creutzfeldt–Jakob disease	27 (0.3)
Gerstmann–Sträussler–Scheinker disease	17 (0.2)
Fatal Familial Insomnia	43 (0.4)
Neurodegenerative	3034 (28.3)
Inflammation	794 (7.4)
Paraneoplastic/CNS tumour	346 (3.2)
Stroke	583 (5.4)
Epileptic fit	218 (2.0)
Psychiatric	459 (4.3)
Metabolic	483 (4.5)
Other	1198 (11.2)
Total	10 731 (100.0)

### Cerebrospinal fluid protein analysis

CSF was taken by lumbar puncture during the clinical investigation of the patient at the notifying hospital and sent to individual laboratories. Tests were conducted in each laboratory according to common agreed standards. CSF was stored at −80°C prior to analysis. CSF was analysed for 14-3-3, tau, phosphorylated tau and amyloid-β_1__–__42_ according to established protocols. Test results were entered into the database.

### Protein 14-3-3 analysis

Protein 14-3-3 was tested in CSF by western blot according to previously published protocols (Hsich *et al.*, 1996; Zerr *et al.*, 1998). Each western blot was analysed with a semi-quantitative method (negative = no signal/band present, positive = strong signal/band present, trace = weak signal/band present). Each blot was run with a positive and negative control. As a positive standard, recombinant 14-3-3 [14-3-3 beta protein (human), AbFrontier Co., Ltd] was used.

### Tau

CSF tau protein was quantitatively analysed using a commercially available ELISA kit according to manufacturer’s instruction (INNOTEST® hTAU Ag, Innogenetics). A positive tau-test for Creutzfeldt–Jakob disease was considered at a cut-off level >1300 pg/ml (Otto *et al.*, 2002; Sanchez-Juan *et al.*, 2006)

### Phosphorylated tau

Human tau, phosphorylated at Thr181 (phosphorylated tau) was measured quantitatively a with commercially available ELISA kit [INNOTEST® PHOSPHO-TAU(181P), Innogenetics]. A pathological elevated phosphorylated tau level was considered at >61 pg/ml according to manufacturer’s instruction, aimed at the diagnosis of Alzheimer’s disease.

### Amyloid β_1–42_

Amyloid β_1–42_ was detected with a commercially available ELISA kit [INNOTEST® ß- AMYLOID(1–42) Innogenetics] for quantitative analysis. A pathological decreased amyloid β_1–42_ assay was considered at <450 pg/ml according to manufacturer’s instruction, aimed at the diagnosis of Alzheimer’s disease.

### Statistical analyses

Descriptive analyses as well as validity analysis were performed using SPSS 19.0 (IBM SPSS Statistics 19). Reliability was estimated using Stata 11 (StataCorp LP). Inter-laboratory reliability was assessed using Cohen’s kappa for comparisons of two raters (data not shown) and Fleiss’ kappa for comparisons of multiple raters. Agreement was estimated according to Landis and Koch (1977): perfect if kappa >0.8, substantial if 0.8 ≥ kappa > 0.6, moderate if 0.6 ≥ kappa > 0.4, fair if 0.4 ≥ kappa > 0.2, slight if 0.2 ≥ kappa > 0, poor if kappa <0 (Supplementary material).

## Results

In total, CSF analysis of dementia markers (14-3-3, tau, phosphorylated tau, amyloid β_1–42_) were performed on 29 022 samples. The main analysis (patient’s with definite clinical diagnosis) comprised 3616 samples from patients with various forms of transmissible spongiform encephalopathies (3254 samples being from patients with sporadic Creutzfeldt–Jakob disease) and 7115 from control subjects (Table 1).

### Diagnoses of rapid progressive dementias in 14-3-3 test referrals

Table 1 demonstrates the rapid progressive dementia diagnoses in patients with suspected Creutzfeldt–Jakob disease referred for CSF protein 14-3-3 testing between the years 1998 and 2008. Differential diagnoses were divided into: prion disorders (*n=* 3616), primary and secondary forms of neurodegenerative dementia (*n=* 3034, including vascular dementia and normal pressure hydrocephalus) and acute neurological disorders such as stroke, CNS inflammation, CNS tumour or epileptic fit (*n=* 1941). Non-neurological causes were from psychiatric (e.g. pseudo-dementia in depression) or metabolic origin (e.g. hyponatraemia) or related to ‘other diagnosis’ (*n=* 2140). The most frequent differential diagnosis was sporadic Creutzfeldt–Jakob disease with 30.3%, followed by neurodegenerative dementia with 28.3% and patients with ‘other diagnosis’ (11.2%). Regarding acute neurological disorders, the group of CNS inflammation was most frequent with 7.4%, followed by stroke (5.4%) and paraneoplastic/CNS tumour (3.2%). Rapid cognitive disturbance from a non-neurological cause comprised 4.3% psychiatric and 4.5% metabolic origin. If we exclude all patients with transmissible spongiform encephalopathies from the analysis, the distribution was as follows: 42.6% neurodegenerative dementia, 27.3% acute neurological disease (most frequently: 11.2% CNS inflammation, 8.2% stroke) and 13.2% non-neurological, potentially treatable cognitive deterioration (6.8% metabolic and 6.4% psychiatric disease).

### Cerebrospinal fluid dementia markers in rapid progressive dementias

**Figure 1:**
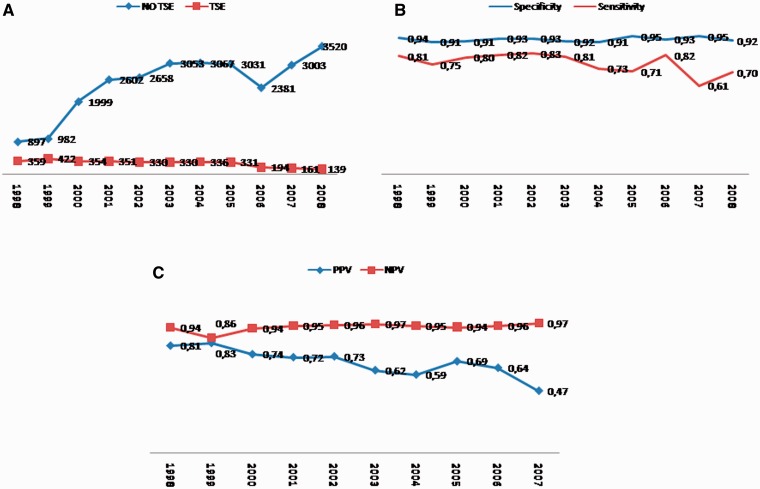
(**A**) Total CSF samples tested by years [blue = no transmissible spongiform encephalopathy (TSE) diagnosis; red = transmissible spongiform encephalopathy diagnosis] from all participating countries. (**B**) Protein14-3-3 specificity and sensitivity by years. (**C**) Protein14-3-3 positive and negative predictive values (PPV/NPV) by years. Of note, decrease in positive predictive values following the increasing numbers of patients with non-transmissible spongiform encephalopathies tested. Negative predictive value remains stable.

In the individual reference laboratories we observed a trend towards increased 14-3-3 test referrals not only for Creutzfeldt–Jakob disease, but also in screening of CSF dementia markers in patients with rapid progressive dementia (Fig. 1A). Going along with this finding, we observed a decrease in our positive predictive values over time. The negative predictive value remains stable (Fig. 1C) Because of this trend, we were able to collect substantial data on dementia markers in these patients. Hereby, we analysed tau, phosphorylated tau and amyloid-β_1__–__42_ according to subgroups of neurodegenerative, acute neurological or non-neurological (potentially reversible) origin in a similar fashion to 14-3-3.

Table 2 summarizes collective data on 14-3-3, tau, phosphorylated tau and amyloid-β_1__–__42_. As previously described, 14-3-3 was negative in 93% of all patients with neurodegenerative disorders. The test became more often false positive in acute neurological events (inflammation 19.2%, stroke 15.2%, epileptic fits 17% and CNS tumour 18.4%) causing a drop in specificity than neurodegenerative and non-neurological forms of dementia. With respect to tau we observed a similar trend. Levels at a cut-off >1300 pg/ml are considered highly suggestive for Creutzfeldt–Jakob disease (Otto *et al.*, 2002; Sanchez-Juan *et al.*, 2006) and were referred to as ‘tau positive’ in our analysis. ‘Tau negative’ results were obtained in 95% of all neurodegenerative diseases. ‘Tau positive’ results were similarly obtained as 14-3-3 in patients with epileptic fits (24%), CNS tumour/paraneoplastic (22%) and stroke (18.4%).

**Table 2:** Overview on 14-3-3 and levels of tau, phosphorylated tau, amyloid β_1–42_ in differential diagnosis of rapid dementia (excluding Creutzfeldt–Jakob disease)

Diagnosis	CSF all, *n*	14-3-3, *n*	14-3-3			Tau, *n*	Tau (pg/ml), median (min–max), positivity (%)	Phosphorylated tau, *n*	Phosphorylated tau (pg/ml), median (min–max)	Amyloid β_1–42_, *n*	Amyloid β_1–42 _(pg/ml), median (min–max)
			Negative (%)	Positive (%)	Trace (%)						
Neurodegenerative	3034	3009	2794/3009 (92.9)	142/3009 (4.7)	73/3009 (2.4)	375	224 (0–38 400) (5.1)	183	55 (10–202)	303	413 (55–1450)
Inflammation	794	785	601/785 (76.6)	151/785 (19.2)	33/785 (4.2)	93	221 (45–3156) (8.6)	13	38 (15–80)	41	491 (115–1094)
Paraneoplastic/CNS tumour	346	342	256/342 (74.9)	63/342 (18.4)	23/342 (6.70)	41	396 (70–29 000) (22)	12	37.5 (20–80)	24	474 (80–878)
Stroke	583	574	465/574 (81)	87/574 (15.2)	22/574 (3.60)	114	269 (41–14 659) (18.4)	37	49 (13–124)	96	478 (80–1481)
Epileptic fit	218	218	171/218 (78.4)	37/218 (17)	10/218 (4.60)	33	329 (75–5584) (24.2)	8	64.5 (20–132)	21	519 (217–1049)
Psychiatric	459	459	439/459 (95.6)	13/459 (2.8)	7/459 (1.5)	63	127 (0–1112) (0)	27	46 (16–215)	60	607 (77–1296)
Metabolic	483	481	438/481 (91.1)	36/481 (7.5)	7/481 (1.5)	42	188 (75–3472) (2.4)	17	39 (12–101)	40	418 (80–1494)

For phosphorylated tau and amyloid-β_1__–__42_ no particular cut-off points are established in Creutzfeldt–Jakob disease diagnosis. For this reason calculation of ‘false positive’ or ‘false negative’ results in the differentiation of Creutzfeldt–Jakob disease from other rapid dementia diagnoses was not possible. However patients with low amyloid-β_1__–__42_ and high phosphorylated tau levels are at a clear risk of Alzheimer’s disease (Hertze *et al.* 2010). We observed a pathologically elevated phosphorylated tau median in the group with epileptic fits (64.5 pg/ml) only. For amyloid-β_1__–__42_, we found pathologically decreased levels in the group of neurodegenerative CNS diseases (413 pg/ml). This was expected as it represents a typical result in patients with Alzheimer’s disease (see later). Of interest, in non-neurological patients with the diagnosis ‘metabolic disorders’ we also detected decreased median levels of amyloid-β_1__–__42_ (418 pg/ml).

### Cerebrospinal fluid dementia markers in neurodegenerative diseases, vascular dementia and normal pressure hydrocephalus

**Figure 2:**
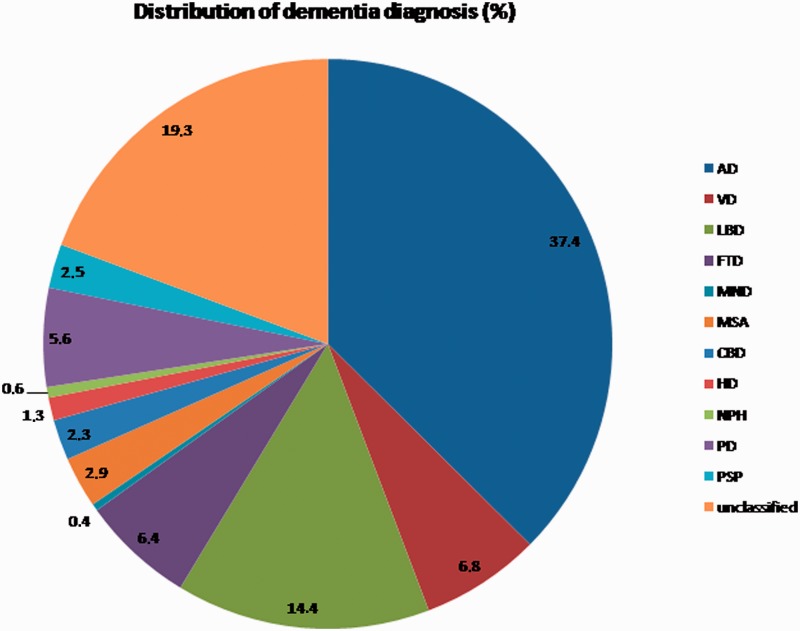
Distribution of neurodegenerative dementia diagnosis. AD = Alzheimer’s disease; CBD = corticobasal degeneration; FTD = frontotemporal dementia; HD = Huntington’s disease; LBD = Lewy body disease; MND = motor neuron disease; MSA = multisystem atrophy; PD = Parkinson’s disease; PSP = progressive supranuclear palsy; NPH = normal pressure hydrocephalus; VD = vascular dementia.

As predetermined cut-off values are not available for the majority of differential diagnoses in this study, the next analysis describes results of CSF dementia marker profiles in various neurodegenerative dementias. Data on CSF dementia markers (14-3-3, tau, phosphorylated tau and amyloid-β_1__–__42_) were evaluated in a total of 3034 patients (28.3% of the total group) with a neurodegenerative disease diagnosis. Out of these patients, 941 (37.4%) displayed a diagnosis of Alzheimer’s disease, followed by 486 patients (19.3%) with unclassified dementia, 362 patients with Lewy body disease (14.4.%), 172 patients with vascular dementia (6.8%), 162 patients with frontotemporal dementia (6.4%), 142 patients with Parkinson’s disease (5.6%), 74 patients with multisystem atrophy (2.9%), 62 patients with progressive supranuclear palsy (2.5%), 57 patients with corticobasal degeneration (2.3%), 33 patients with Huntington’s disease (1.3%), 16 patients with normal pressure hydrocephalus (0.6%) and 10 patients with motor neuron disease (0.4%) (Fig. 2).

A positive 14-3-3 test was seen in patients with vascular dementia (10.7%) followed by Alzheimer’s disease (5.8%) and Lewy body disease (5.3%) (Table 3). A ‘positive tau’ (at Creutzfeldt–Jakob disease cut-off >1300 pg/ml) was obtained in vascular dementia (7.8%), Alzheimer’s disease (7.6%), Parkinson’s disease (2.0%) and Lewy body disease (1.8%) (Table 4). The median tau ranged highest in the group of Alzheimer’s disease (314 pg/ml) and Huntington’s disease (383 pg/ml) followed by vascular dementia (206 pg/ml) and Lewy body disease (216 pg/ml) (Table 5). Median phosphorylated tau levels of other neurodegenerative dementias ranged at normal levels; however, all neurodegenerative disease groups displayed pathologically elevated phosphorylated tau levels at maximum range. For amyloid-β_1__–__42_, pathologically decreased levels (<450 pg/ml) were observed in Alzheimer’s disease (median 404 pg/ml), Lewy bodydisease (336 pg/ml) and Huntington’s disease (321 pg/ml) (Table 5).

**Table 3:** Results of positive 14-3-3 tests in neurodegenerative dementia (excluding Creutzfeldt–Jakob disease)

Protein 14-3-3				
Diagnosis	Total *n*	Negative (%)	Positive (%)	Trace (%)
Alzheimer’s disease	932	851 (91.3)	54 (5.8)	27 (2.9)
Vascular dementia	169	144 (82.5)	18 (10.7)	7 (4.1)
Lewy body disease	358	331 (92.5)	19 (5.3)	8 (2.2)
Frontotemporal dementia	162	150 (92.6)	7 (4.3)	5 (3.1)
Motor neuron disease	10	10 (100)	0 (0)	0 (0)
Multisystem atrophy	74	68 (91.6)	2 (2.7)	4 (5.4)
Corticobasal degeneration	57	56 (98.2)	1 (1.8)	0 (0)
Huntington’s disease	32	32 (100)	0 (0)	0 (0)
Normal pressure hydrocephalus	16	15 (93.8)	0 (0)	1 (6.3)
Parkinson’s disease	140	134 (95.7)	6 (4.3)	0 (0)
Progressive supranuclear palsy	62	60 (96.8)	2 (3.2)	0 (0)

**Table 4:** Results of positive tau tests (>1300 pg/ml) in neurodegenerative dementia (excluding Creutzfeldt–Jakob disease)

Tau (cut-off >1300 pg/ml)			
Diagnosis	Total (*n*)	Negative (%)	Positive (%)
Alzheimer’s disease	132	122 (92.4)	10 (7.6)
Vascular dementia	64	59 (92.2)	5 (7.8)
Lewy body disease	55	54 (98.2)	1 (1.8)
Frontotemporal dementia	28	28 (100)	0 (0)
Motor neuron disease	1	1 (100)	0 (0)
Multiple system atrophy	15	15 (100)	0 (0)
Corticobasal degeneration	4	4 (100)	0 (0)
Huntington’s disease	4	4 (100)	0 (0)
Parkinson’s disease	51	50 (98)	1 (2)
Progressive supranuclear palsy	11	10 (90.9)	1 (9.1)
Other dementia	12	12 (100)	0 (0)

**Table 5:** Overview on levels of tau, phosphorylated tau and amyloid β_1–42_ in neurodegenerative dementia (excluding Creutzfeldt–Jakob disease)

Diagnosis	Tau (pg/ml)		Phosphoylated tau (pg/ml)*		Amyloid β_1–42_ (pg/ml)*	
	Total *n*	Median (min–max)	Total *n*	Median (min–max)	Total *n*	Median (min–max)
Alzheimer’s disease	132	314 (75–8766)	51	83 (19–202)	108	404 (80–1445)
Vascular dementia	64	206 (75–4878)	25	49 (17–110)	147	490 (80–1481)
Lewy body disease	55	214 (75–1395)	35	54 (10–182)	327	336 (89–1450)
Frontotemporal dementia	28	159 (70–554)	16	39 (18–105)	146	497 (110–865)
Multiple system atrophy	15	136 (70–740)	7	41 (17–72)	67	481 (93–852)
Corticobasal degeneration	4	219 (164–389)	3	60 (53–65)	54	457 (282–549)
Huntington’s disease	4	383 (75–933)	3	31 (20–123)	30	321 (222–1419)
Parkinson’s disease	51	179 (0–756)	37	55 (17–106)	105	476 (80–1304)
Progressive supranuclear palsy	11	131 (75–275)	9	38 (11–69)	53	524 (77–756)

**Figure 3:**
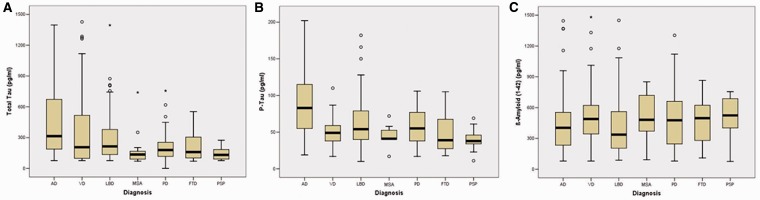
(**A**) Boxplot of tau levels. (**B**) Boxplot of phosphorylated tau (P-Tau) levels. (**C**) Boxplot of amyloid-β_1–42_ levels in neurodegenerative dementia diagnosis. AD = Alzheimer’s disease; FTD = frontotemporal dementia; LBD = Lewy body disease; MSA = multisystem atrophy; PD = Parkinson’s disease; PSP = progressive supranuclear palsy; VD = vascular dementia.

To illustrate our findings, we have grouped diseases according to similar pathology: (i) Alzheimer’s disease, (ii) vascular dementia, (iii) α-synucleinopathies including Parkinson’s disease, Lewy body disease and multisystem atrophy and (iv) tauopathies including frontotemporal dementia and progressive supranuclear palsy (Fig. 3A–C). Here we can show that median levels of tau and phosphorylated tau generally range higher in Alzheimer’s disease, vascular dementia and Lewy body disease whereby in contrast, median levels of amyloid-β_1__–__42_ are generally reduced in patients with Alzheimer’s disease and patients with Lewy body disease when compared with other disease groups here.

### Cerebrospinal fluid test referrals per year

We observed a constant increase in CSF test referrals per year between 1998 and 2008 that was seen in all participating countries (Fig. 1A). In spite of the increase in CSF tests, there was no increase in the total number of cases with identified transmissible spongiform encephalopathies. In the years 1998–2005 we identified between 330 and 422 cases with transmissible spongiform encephalopathies per year. Between 2005 and 2008, the number of cases with transmissible spongiform encephalopathies appeared reduced (139–194 cases per year), because collective data was not available from all participating countries during this time period.

### Cerebrospinal fluid 14-3-3 specificity in sporadic Creutzfeldt–Jakob disease

Despite of the increasing number of CSF referrals per year for detection of 14-3-3 in suspected cases of Creutzfeldt–Jakob disease in the 10-year period, we calculated a stable and high specificity of 14-3-3 that ranged from 0.91 to 0.95 per year (Fig. 1B). The total 14-3-3 specificity for Creutzfeldt–Jakob disease during the observed time period was 0.92. When compared with all patients with definite diagnoses only, the specificity was slightly lower (0.90).

### Influence of 14-3-3 specificity by differential diagnosis

**Figure 4:**
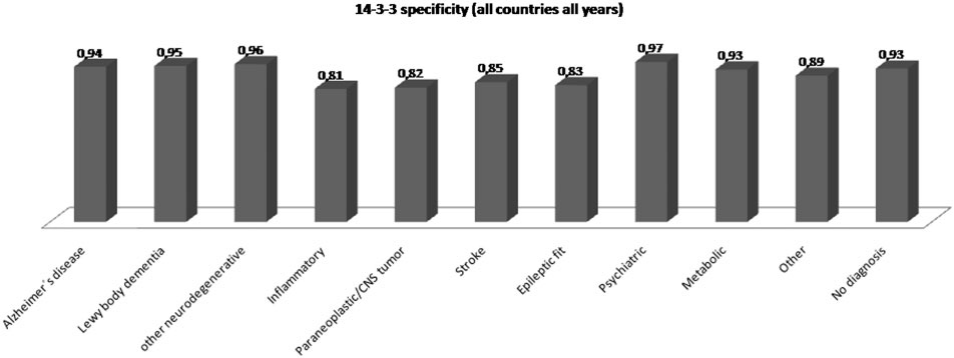
Protein 14-3-3 specificity stratified by differential diagnosis (this includes total number of 14-3-3 tests in all countries all years). See also Table 6.

The overall test specificity varied with respect to differential diagnosis. A lower 14-3-3 specificity was observed in discrimination to acute neurological events (82–87%), a high 14-3-3 specificity was obtained in neurodegenerative diseases (95–97%) and non-neurological conditions (91–97%) (Fig. 4 and Table 6).

**Table 6:** Protein 14-3-3 specificity stratified by differential diagnosis (this includes total number of 14-3-3 tests in all countries all years)

Diagnostic category	Negative/ trace (*n*)	Positive (*n*)	Total (*n*)	Specificity
Alzheimer’s disease	878	54	932	0.94
Lewy body dementia	339	19	358	0.95
Other neurodegenerative	1156	51	1207	0.96
Inflammatory	634	151	785	0.81
Paraneoplastic/CNS tumour	279	63	342	0.82
Stroke	487	87	574	0.85
Epileptic fit	181	37	218	0.83
Psychiatric	446	13	459	0.97
Metabolic	445	36	481	0.93
Other	1046	132	1178	0.89
No diagnosis	16 824	1248	18 072	0.93
Total	22 715	1891	24 606	0.92

See also Fig. 4.

## Discussion

### Rapid progressive dementia diagnosis

The spectrum of differential diagnosis of rapid dementia ranged from neurodegenerative, acute neurological (e.g. CNS inflammation, tumour, stroke) and non-neurological (e.g. psychiatric, metabolic) origin. The most frequent neurodegenerative disease diagnoses in our cohort were Alzheimer’s disease, Lewy body disease and vascular dementia. This follows a similar trend when compared to previous reports from longitudinal national surveillance studies (Van Everbroeck *et al.*, 2004; Heinemann *et al.*, 2007; Josephs *et al.*, 2009; Chitravas *et al.*, 2011). Likewise, we observed a significant ratio of potentially treatable forms of rapid dementia diagnoses that were previously identified as important differential diagnoses of suspected transmissible spongiform encephalopathies (Chitravas *et al.* 2011).

### Results of dementia markers in neurodegenerative dementias and rapid dementia from acute neurological diseases

Many studies on amyloid β_1__–__42_, total tau levels and its phosphorylated isoforms have been performed in CSF biomarker-supported diagnosis of Alzheimer’s disease. The combined measurement of tau and amyloid β_1__–__42_ in CSF has proven diagnostic accuracy for Alzheimer’s disease (Hulstaert *et al.*, 1999). However, in most studies, the number of patients per group is limited and various criteria and diagnostic techniques have been applied (Zerr *et al.*, 2011). Amyloid β_1__–__42_ levels are decreased in patients with Alzheimer’s disease, but might also decrease in other types of dementia. Test sensitivity for amyloid β_1__–__42_ alone is given from 60–96%, depending on the design of the study (Zerr *et al.* 2011). Elevated levels of tau and phoshorylated tau are recognized hallmarks in the CSF supported diagnosis of Alzheimer’s disease (Kapaki *et al.*, 2007; Snider *et al.*, 2009; Hertze *et al.*, 2010; Scheurich *et al.* 2010; van Harten *et al.*, 2011).

Compared with Alzheimer’s disease, elevated tau or reduced amyloid-β_1__–__42_ are usually not found in other forms of neurodegenerative dementia (e.g. tauopathies, vascular dementia, Lewy body disease) (Grossman *et al.*, 2005; Hu *et al.*, 2010). A recent large US cohort study analysed CSF dementia marker profiles on neurodegenerative dementia (Schoonenboom *et al.*, 2012) and obtained similar results as the study presented here. A profile as seen in Alzheimer’s disease was detected in patients with Lewy body disease (47%), corticobasal degeneration (38%), vascular dementia and frontotemporal dementia (both ∼30%). When analysing median CSF levels for tau and amyloid β_1__–__42_ alone, all above mentioned groups ranged at normal values, thus their findings are corresponding well to our results both in median levels and minimum/maximum values.

When focusing on CSF markers in rapid dementia of acute neurological origin data in the literature are limited. As inflammatory diseases are potentially treatable, the differential diagnosis of this group is of special importance. With respect to the biomarker profiles, these patients had less frequently increased tau levels in CSF (8.6%) than a positive 14-3-3 test (19.2%). Thus, in particular circumstances, high 14-3-3 levels and at the same time low tau levels in the CSF might be indicative for a potentially treatable inflammatory or autoimmune mediated disorder.

### Specificity of protein 14-3-3

A positive CSF 14-3-3 test displays one of the important diagnostic tools in the identification of patients with Creutzfeldt–Jakob disease at present because from technical point of view this marker is stable and CSF samples can be forwarded for testing to specialized laboratories. Meanwhile, the test is widely available and serves as an important tool in surveillance studies worldwide. Because test specificity was argued to be low based on single centre reports and small selected cohorts, our goal was to analyse 14-3-3 specificity in a 10-year longitudinal multicentre study in a large unbiased cohort of patients.

A large number of studies proved that in the appropriate clinical circumstances a positive 14-3-3 test is highly sensitive and specific for sporadic Creutzfeldt–Jakob disease diagnosis. Protein 14-3-3 detection correlated with clinical diagnosis in 85–94% in sporadic Creutzfeldt–Jakob disease. Specificities ranging from 84% (Zerr *et al.*, 2000*a*) and 85% (Sanchez-Juan *et al.*, 2007) to 100% (immunoblot; Beaudry *et al.*, 1999) and immunoassay (cut-off >8 ng/ml; Aksamit *et al.*, 2001) have been reported. The majority of studies on national and multicentre levels reported a 14-3-3 specificity >90% (Hsich *et al.*, 1996; Zerr *et al.*, 1998; Lemstra *et al.*, 2000; Van Everbroeck *et al.*, 2003; Cuadrado-Corrales *et al.*, 2006; Green *et al.*, 2007). However, with increasing numbers of test referrals per year, we observed that a number of positive tests were related to non-Creutzfeldt–Jakob disease diagnosis mostly of acute neurological origin. This observation was supposed to impact on previously reported high specificity and subsequently led to question the value of this test in Creutzfeldt–Jakob disease diagnosis (Perry and Geschwind 2011).

With our analysis we provide evidence that in spite of increasing CSF tests per year—a phenomena experienced in all participating countries—the specificity of 14-3-3 remained highly stable an overall specificity of 92%. The number of patients with Creutzfeldt–Jakob disease diagnosis increased with time after this test became available but there was no further substantial increase in numbers after 2000.

Rapid dementia caused by acute neurological events (e.g. inflammation, stroke, CNS tumour or epileptic fits) is the main reason for a drop in test specificity from 92% to 82–85%. It is not clear why such CSF samples were referred for 14-3-3 testing since these conditions do not represent a Creutzfeldt–Jakob disease differential diagnosis. If tested in the context of neurodegenerative disease alone, the test specificity was high (95–97%). A careful interpretation of a positive 14-3-3 test in the clinical context is therefore mandatory to exclude acute neurological events which is easily achieved taken together information on basic CSF results (e.g. cell count, total protein and oligoclonal bands) as well as imaging results (exclusion of tumour, encephalitis and stroke) and EEG (epileptic pattern) together with the thorough ascertainment of the patient’s clinical history.

Recent studies from UK and Canadian cohorts, however, reported discrepant lower results on 14-3-3 specificities ranging from 72% (Coulthart *et al.*, 2011) to 74% (Chohan *et al.*, 2010). Previous studies, including an earlier analysis of our multicentre consortium, identified influencing parameters on 14-3-3 test specificity such as time point of CSF analysis during disease course, thorough diagnostic work-up of related differential diagnoses and *PRNP* 129 genotype (Zerr *et al.*, 2000*b*; Sanchez-Juan *et al.*, 2006; Pennington *et al.*, 2009). In this view, it is of consideration whether the reported lower 14-3-3 specificities were due to single centred cohorts or heterogeneous analysis of 14-3-3 results with regard to above mentioned influencing factors (e.g. clarification/follow up of disease diagnosis or timing). Also potential reasons might be differences in test methods (western blot versus immunoassay), variations in test protocols and verification of 14-3-3 results in ring trials in different centres.

In our study, we provide results on ‘total’ 14-3-3 specificity calculated on almost 30 000 CSF analyses. For various reasons (e.g. no information, patients lost at follow up and data protection regulations), the final diagnosis was not always available. We are aware that this might result in a selection bias in relation to the patients where a diagnosis was achieved. On the other hand, definite diagnoses of non-Creutzfeldt–Jakob disease cases in national reference centres are usually only obtained if the respective patient is seriously suspected to suffer from Creutzfeldt–Jakob disease. This is much more likely, when 14-3-3 testing shows a (false) positive result. The respective group with a definite diagnosis might therefore be even more prone to selection bias than a control group including all patients reported to the reference centres. Therefore we provided all figures to demonstrate that specificity figures were not affected much by this approach (total specificity 92%, in definite diagnoses specificity: 81–97%).

## Conclusion

In conclusion, our multicentre longitudinal study demonstrates that CSF protein 14-3-3 detection remains an important test in the diagnosis of Creutzfeldt–Jakob disease. Due to a loss in specificity in acute neurological events, the interpretation of positive 14-3-3 results needs to be performed in the clinical context. The spectrum of differential diagnosis of rapid progressive dementia included those of neurodegenerative origin, dementia in acute neurological diseases as well as from non-neurological origin. Thus, a thorough clinical work-up of rapid dementia diagnosis should be performed to identify their origin especially with regard of potentially treatable forms. CSF biomarkers are helpful in differentiation of rapid dementia forms such as Creutzfeldt–Jakob disease, but also in solid identification of patients with Alzheimer’s disease. Furthermore, in some dementia due to inflammation, where a 14-3-3 test might be false positive, low levels of tau might be helpful in discriminating forms of neurodegenerative dementia.

## Funding

This work was supported by grants from the European Commission (CJDmarkers (Early clinical diagnosis of human spongiform encephalopathies by analysis of biological fluids, QLG3-CT-2002-81606), PRIORITY (Protecting the food chain from prions: shaping European priorities through basic and applied research, FP7-KBBE-2007-2A, project number 222887) DEMTEST (Biomarker based diagnosis of rapid progressive dementias-optimization of diagnostic protocols, 01ED1201A)).

## Supplementary material

Supplementary material is available at *Brain* online.

Supplementary Data
