# Supplementary material for: Cerebrospinal fluid biomarker supported diagnosis of Creutzfeldt–Jakob disease and rapid dementias: a longitudinal multicentre study over 10 years
Source: Brain. 2012 Sep 25;135(10):3051–61. doi: 10.1093/brain/aws238 (PMC3470713; doi:10.1093/brain/aws238)
Supplement: Supplementary Data [file supp_135_10_3051__index.html]

Supplementary Data 

# Cerebrospinal fluid biomarker supported diagnosis of Creutzfeldt–Jakob disease and rapid dementias: a longitudinal multicentre study over 10 years

## Supplementary Data

files

**Files in this Data Supplement:**

- Supplementary Data - doc file
- Supplementary Data - doc file
