# Supplementary material for: Cerebrospinal fluid biomarker supported diagnosis of Creutzfeldt–Jakob disease and rapid dementias: a longitudinal multicentre study over 10 years
Source: Brain. 2012 Sep 25;135(10):3051–61. doi: 10.1093/brain/aws238 (PMC3470713; doi:10.1093/brain/aws238)
Supplement: Supplementary Data [file supp_aws238_material.doc]

**Supplementary: Ring trials for 14-3-3 inter-laboratory reliability**

Methods. Four ring trials were conducted between participating laboratories. Samples were derived from patients with CJD (positive sample), healthy controls (negative sample) and patients with other diagnoses of neurodegenerative or non-neurodegenerative origin (negative/trace samples) or samples achieving atypical profiles (double bands, e.g. as seen in inflammation, blood contamination). Test results were qualitatively assessed as positive, trace, negative or atypical profile. Inter-laboratory reliability was analysed.

Results. Moderate agreement could be achieved in 3 of 4 ring trials with slightly increasing reliability in more recent years when a consensus decision was made on how to handle inconclusive laboratory results. (Table Supplementary 3)

Disagreement was observed more frequently in samples which were estimated as slightly positive (trace) or with atypical profile by one or more laboratories and which were neither CJD cases nor healthy controls.

***Discussion.*** Results of inter-laboratory reliability show moderate agreement only. Fleiss kappa is a conservative measure for inter-reader agreement. Nevertheless, it was expected that agreement within a European network of reference laboratories was higher than observed. Reasons might be that despite standardized protocols undetected differences in test kits and methods might be present. Besides, for the ring trials reported in this study respective CSF samples were not a random sample of all CSF samples received by the reference centers for 14-3-3 testing. Atypical and borderline 14-3-3 samples were overrepresented whereas negative and positive samples were underrepresented. Samples in which agreement could not be achieved had either an atypical profile or were only weak positive. On the one hand, inter-laboratory reliability was thereby biased towards 0. On the other hand, this suggests that there are still severe differences in the interpretation of borderline positive (trace, weak positive) and atypical 14-3-3 results.

Further analysis of the reasons of observed kappa values is necessary and has been initiated. Better agreement must be achieved especially for borderline samples.

The moderate inter-laboratory reliability seen in this study must be regarded as a limitation of the presented results. Interpretation of the results of this study and further studies using the respective data must respect this and should be performed with caution.

**Table Supplementary :** Results of four ring trials (Fleiss kappa): Moderate agreement could be achieved in 3 of 4 ring trials with slightly increasing reliability in more recent years when a consensus decision was made on how to handle inconclusive lab results.

| **year (number of participants)** | **Fleiss’ kappa** |
| --- | --- |
| **2003 (n=3)** | 0.68 |
| **2004 (n=5)** | 0.40 |
| **2008 (n=7)** | 0.54 |
| **2009 (n=7)** | 0.55 |
